# Influence of Occlusal Hypofunction on Alveolar Bone Healing in Rats

**DOI:** 10.3390/ijms24119744

**Published:** 2023-06-05

**Authors:** Anna Damanaki, Svenja Beisel-Memmert, Marjan Nokhbehsaim, Ali Abedi, Birgit Rath-Deschner, Andressa V. B. Nogueira, James Deschner

**Affiliations:** 1 Department of Periodontology and Operative Dentistry, University Medical Center, University of Mainz, 55131 Mainz, Germany; 2 Department of Orthodontics, Center of Dento-Maxillo-Facial Medicine, University of Bonn, 53111 Bonn, Germany; 3 Section of Experimental Dento-Maxillo-Facial Medicine, Center of Dento-Maxillo-Facial Medicine, University of Bonn, 53111 Bonn, Germany

**Keywords:** bone healing, occlusal hypofunction, fenestration, animals, periodontitis

## Abstract

The aim of this in vivo study was to investigate the effect of occlusal hypofunction on alveolar bone healing in the absence or presence of an enamel matrix derivative (EMD). A standardized fenestration defect over the root of the mandibular first molar in 15 Wistar rats was created. Occlusal hypofunction was induced by extraction of the antagonist. Regenerative therapy was performed by applying EMD to the fenestration defect. The following three groups were established: (a) normal occlusion without EMD treatment, (b) occlusal hypofunction without EMD treatment, and (c) occlusal hypofunction with EMD treatment. After four weeks, all animals were sacrificed, and histological (hematoxylin and eosin, tartrate-resistant acid phosphatase) as well as immunohistochemical analyses (periostin, osteopontin, osteocalcin) were performed. The occlusal hypofunction group showed delayed bone regeneration compared to the group with normal occlusion. The application of EMD could partially, but not completely, compensate for the inhibitory effects of occlusal hypofunction on bone healing, as evidenced by hematoxylin and eosin and immunohistochemistry for the aforementioned molecules. Our results suggest that normal occlusal loading, but not occlusal hypofunction, is beneficial to alveolar bone healing. Adequate occlusal loading appears to be as advantageous for alveolar bone healing as the regenerative potential of EMD.

## 1. Introduction

Periodontitis is a chronic inflammatory disease caused by a dysbiotic microbiome [1,2]. In this dysbiotic periodontal environment, various microorganisms trigger inflammatory processes that lead to the destruction of periodontal tissues [1,2]. Several local and systemic co-factors contribute to the development and progression of periodontitis. For example, smoking, genetic predisposition, and various systemic diseases, e.g., diabetes mellitus, are known to promote the development of periodontitis [3,4,5,6,7,8,9]. Periodontitis is one of the most common diseases and its prevalence is increasing worldwide due to rising life expectancy, multimorbidity, and tooth preservation [10,11,12,13,14]. It is characterized by bone and attachment loss and can lead to tooth loss if left untreated [2]. Socioeconomic disadvantages such as lack of health insurance, low levels of education, poverty, and lack of access to health care lead to more severe periodontal disease and, thus, a higher number of teeth being extracted [15,16,17]. Stage IV periodontitis is associated with multiple tooth loss, tooth hypermobility, posterior bite collapse, and/or masticatory dysfunction [18,19]. If the tooth loss caused by periodontitis is not compensated by prosthetic, implant, and/or orthodontic rehabilitation, hypofunction or hyperfunction may occur in some teeth [19].

The aim of periodontal treatment is to reduce the dysbiotic biofilm, and thus stop or delay further destruction of the periodontal soft and hard tissues [20]. This is primarily achieved by subgingival instrumentation using hand- and power-driven instruments, and in rare cases completed by antibiosis. Very advanced cases may require additional surgical intervention, which usually leads to reparative healing [20,21,22]. When the defect morphology permits, regenerative therapy procedures with enamel matrix derivative (EMD), graft materials, and/or membranes, are also frequently used [22,23]. This periodontal regeneration then ideally leads to the restoration of the form, structure, and function of the lost periodontal tissues [22,23].

Enamel matrix proteins are molecules deposited on the root surface during odontogenesis by cells of the Hertwig’s epithelial root sheath prior to cementum formation, which provides the stimulus for cementogenesis [24]. Commercially available enamel matrix proteins, i.e., EMD, for periodontal therapy are derived from porcine tooth buds, consist of 90% amelogenins, and are applied during periodontal surgery [24]. Several in vitro, in vivo and human histological studies have demonstrated that treatment with EMD promotes new bone formation [24]. For example, the use of EMD, either alone or in combination with membranes and grafts, in the treatment of intrabony defects and Class II furcation defects has been shown to improve the outcomes in several meta-analyses [25,26,27]. Human histological studies confirmed that EMD promotes regenerative healing [28,29,30]. Some studies have shown that the effect of EMD depends on the environmental conditions, such as inflammation, microorganisms, nicotine, glucose, adiposity, and mechanical stress [31,32,33,34,35,36,37,38,39].

Several studies have shown that EMD regulates key cell functions, such as adhesion, spreading, proliferation, differentiation and survival. These effects of EMD are mediated at molecular levels by the regulation of transcription and growth factors, cytokines, extracellular matrix molecules and factors involved in bone remodeling [24]. For example, it has been demonstrated that EMD promotes the expression and protein synthesis of transforming growth factor-β1, insulin-like growth factor, bone morphogenetic protein-2 (BMP-2), vascular endothelial growth factor and connective tissue growth factor in several cell types [31,40,41,42]. Moreover, EMD stimulated the production of collagen type I and periostin in periodontal cells [31,41,43]. The deposition of calcium was also enhanced by the incubation of osteoblast-like cells with EMD [31,41]. Furthermore, EMD has been shown to modulate wound healing by promoting both soft tissue regeneration and angiogenic activity [31,41]. It is thought that EMD uses different intracellular pathways for its beneficial effects, such as the SMAD and MAPK pathways [44,45,46,47]. Interestingly, the positive effects of EMD seem to be reduced in an inflammatory, microbial or biomechanical environment [31,39,41,48,49]. For example, our in vitro studies revealed that the EMD-induced production of growth factors and matrix molecules, as well as the mineralization, is compromised in the presence of biomechanical loading or proinflammatory mediators [31,39,41]. Interestingly, EMD also seems to exert anti-inflammatory effects [31]. Although EMD also has an antimicrobial influence on cells, it is obvious that these antibacterial effects result from the vehicle of the commercially available product [48,49]. In addition, tobacco smoke also seems to counteract the regenerative effects of EMD [33].

To date, little is known about the effects of occlusal hypofunction, e.g., due to the absence of an antagonistic tooth after extraction of periodontal hopeless teeth, on the regenerative effects of EMD. It has been shown that the missing stimulatory effects of occlusion lead to compromised alveolar and jaw bone formation [50]. Bone remodeling involves the formation of bone as well as bone resorption, with osteoclasts playing a crucial role in this process. Tartrate-resistant acid phosphatases (TRAPs) are a class of metalloenzymes used as markers for osteoclasts [51]. Under normal occlusal conditions, the alveolar bone undergoes continuous bone remodeling to adapt to mechanical stress and to repair microdamage [52,53]. In cases of masticatory overload, the number of TRAP-positive cells is higher than in normal occlusion [54,55,56]. Several molecules, such as periostin, osteopontin and osteocalcin, are involved in bone homeostasis and regeneration [57,58,59] and affected by the cellular environment, such as inflammation [60,61] and mechanical forces [56,60]. Periostin seems to be involved in bone loss due to the lack of balanced bone resorption and remodeling under the conditions of mechanical unloading, such as occlusal hypofunction [60]. A molecule that also plays a role in the bone remodeling caused by mechanical stress is osteopontin [56,62]. Another molecule that plays an important role in bone remodeling is osteocalcin. Osteocalcin binds closely to hydroxyapatite and forms a complex with collagen via osteopontin. Osteocalcin is thus thought to form a bridge between the matrix and the inorganic fraction of bone tissue [59]. Periostin, osteopontin, and osteocalcin thus appear to be closely linked proteins [60]. Conceivably, hypofunction may promote the healing process, such as for splinting after a bone fracture. On the other hand, important stimulatory biomechanical stimuli for regenerative processes might be missing. Therefore, the aim of this in vivo study was to investigate the effect of occlusal hypofunction on alveolar bone healing in the absence and presence of EMD in an animal model.

## 2. Results

### 2.1. Effect of Occlusal Hypofunction on Alveolar Bone Healing

**Figure 1:**
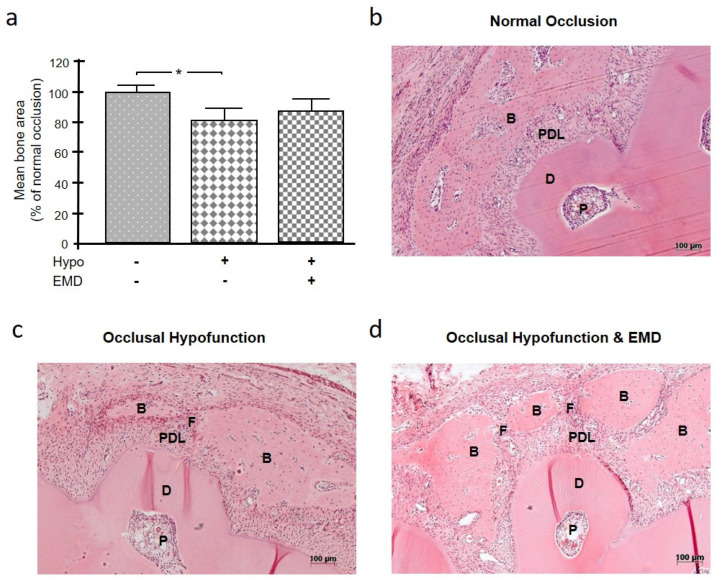
Effect of occlusal hypofunction on alveolar bone healing in the presence and absence of EMD (**a**). A representative image of H&E-stained tissue sections from each group is shown (**b**–**d**). Bars show mean ± SEM; n = 5 animals/group; * significant (*p* < 0.05) difference between groups. B (bone), D (dentin), EMD (enamel matrix derivative), F (fenestration), hypo (occlusal hypofunction), P (pulp), PDL (periodontal ligament).

After a healing period of 4 weeks, it was examined whether occlusal hypofunction exerts an effect on bone healing and, if so, whether this effect is modulated by the application of a regenerative material, i.e., EMD. The animals that lacked the first maxillary molar and therefore suffered from occlusal hypofunction exhibited delayed bone regeneration compared to the normal occlusion group, regardless of the application of EMD (Figure 1a–d). The difference between the normal occlusion group and the occlusal hypofunction group that was not treated with EMD was significant (*p* = 0.037) (Figure 1a). The application of EMD could partially compensate for the negative effect of occlusal hypofunction on bone healing so that the difference from the animals with normal occlusion was not significant (Figure 1a). The sub-analysis for the separate levels (coronal, middle, apical) showed that the bone formation was lowest at the coronal level in occlusal hypofunction with or without EMD. The difference between normal occlusion and occlusal hypofunction without EMD was statistically significant (*p* = 0.048). The data are shown in Figure S1 in the Supplementary Material.

### 2.2. Influence of Occlusal Hypofunction on the Number of TRAP+ Cells

**Figure 2:**
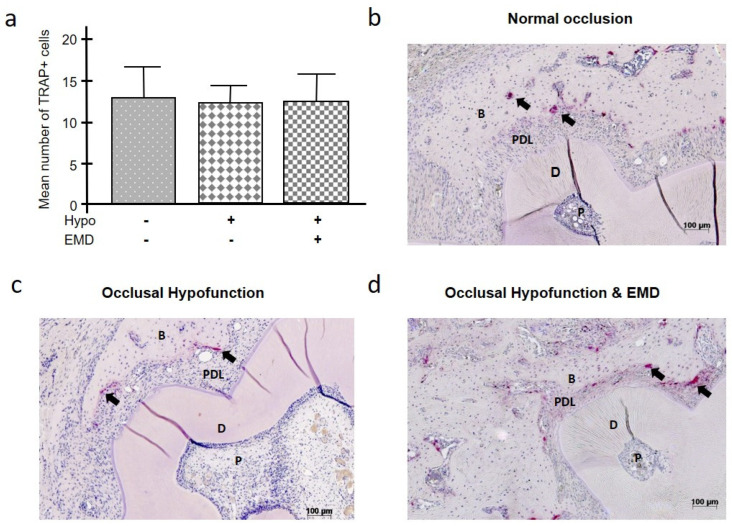
Impact of occlusal hypofunction on the number of osteoclasts in the presence and absence of EMD (**a**). A representative image of tissue sections stained for TRAP from each group is shown (**b**–**d**). TRAP+ cells are indicated by black arrows (**b**–**d**). Bars show mean ± SEM; n = 5 animals/group; B (bone), D (dentin), EMD (enamel matrix derivative), hypo (occlusal hypofunction), P (pulp), PDL (periodontal ligament), TRAP (tartrate-resistant acid phosphatase).

Osteoclasts were visualized by staining for TRAP as a marker of osteoclast activity, and thus bone resorption and remodeling. As shown in Figure 2a–d, the presence of TRAP+ cells was similar in all groups. The statistical analysis failed to reveal any significant differences between the groups.

### 2.3. Effect of Occlusal Hypofunction on Periostin, Osteopontin, and Osteocalcin

Histological sections from each group were stained with different antibodies against molecules associated with bone formation and remodeling, i.e., periostin, osteopontin, and osteocalcin. The intensity of immunohistochemical staining was determined and then assigned to one of five categories (very low, low, moderate, high, very high), which were defined by the respective quintiles.

**Figure 3:**
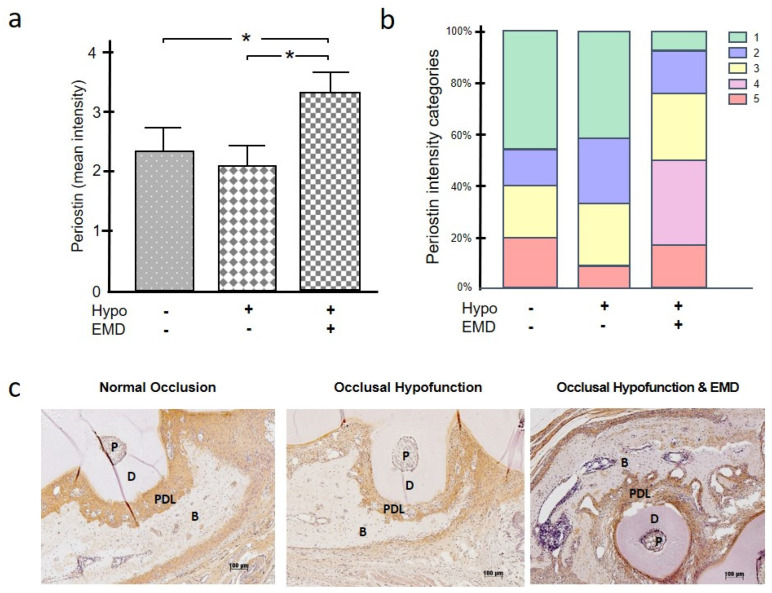
Impact of occlusal hypofunction on synthesis of periostin in the presence and absence of EMD. Mean intensity of periostin (**a**). Frequency distribution of different intensity categories for periostin. The intensity was assigned to five intensity categories (1 = very low, 2 = low, 3 = moderate, 4 = high, 5 = very high) (**b**). A representative immunohistochemistry image from each group is shown (**c**). Bars show mean ± SEM; n = 5 animals/group; * significant (*p* < 0.05) difference between groups. B (bone), D (dentin), EMD (enamel matrix derivative), hypo (occlusal hypofunction), P (pulp), PDL (periodontal ligament).

At the defect sites in occlusal hypofunction without EMD treatment, periostin protein synthesis was lower, although not significantly, than in the defects of the normal occlusion group (Figure 3a–c). The application of EMD could not only compensate for the inhibitory effect of occlusal hypofunction on periostin but even resulted in a greater periostin protein synthesis, with a significant difference compared to the normal occlusion group (*p* = 0.036) and the occlusal hypofunction group without EMD treatment (*p* = 0.011) (Figure 3a). The inhibitory effect of hypofunction and the stimulatory effect of EMD on periostin synthesis were also reflected in the different distribution of intensity categories between the groups. The defects of the normal occlusion group and those of the occlusal hypofunction group without EMD treatment showed low categories more frequently than those of the occlusal hypofunction group treated with EMD (Figure 3b).

**Figure 4:**
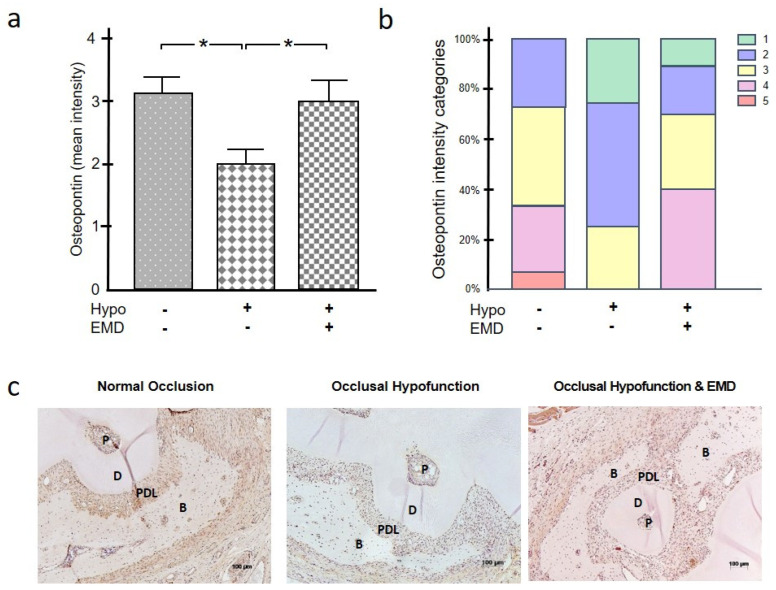
Impact of occlusal hypofunction on synthesis of osteopontin in the presence and absence of EMD. Mean intensity of osteopontin (**a**). Frequency distribution of different intensity categories for osteopontin. The intensity was assigned to five intensity categories (1 = very low, 2 = low, 3 = moderate, 4 = high, 5 = very high) (**b**). A representative immunohistochemistry image from each group is shown (**c**). Bars show mean ± SEM; n = 5 animals/group; * significant (*p* < 0.05) difference between groups. B (bone), D (dentin), EMD (enamel matrix derivative), hypo (occlusal hypofunction), P (pulp), PDL (periodontal ligament).

Osteopontin, a protein involved in the maintenance of bone structure, was also detectable in the histological sections. Occlusal hypofunction resulted in a significantly lower synthesis of osteopontin (*p* = 0.002) (Figure 4a–c). The inhibitory effect of occlusal hypofunction was compensated by the application of EMD, i.e., the intensity of osteopontin staining was significantly stronger in the EMD-treated occlusal hypofunction group (*p* = 0.007) than in the occlusal hypofunction group without EMD application, approaching the level of the normal occlusion group (Figure 4a–c). The distribution pattern of the intensity categories for the individual groups confirmed the negative effect of occlusal hypofunction and the positive influence of EMD. While high and very high osteopontin intensities were observed in normal occlusion and occlusal hypofunction with EMD treatment, only low to moderate intensity categories were found in occlusal hypofunction without EMD application (Figure 4b).

**Figure 5:**
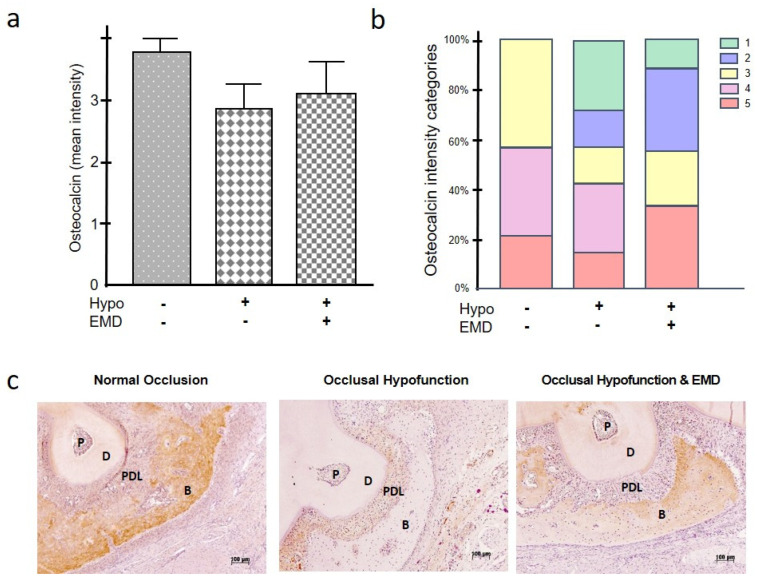
Impact of occlusal hypofunction on synthesis of osteocalcin in the presence and absence of EMD. Mean intensity of osteocalcin (**a**). Frequency distribution of different intensity categories for osteocalcin. The intensity was assigned to five intensity categories (1 = very low, 2 = low, 3 = moderate, 4 = high, 5 = very high) (**b**). A representative immunohistochemistry image from each group is shown (**c**). Bars show mean ± SEM; n = 5 animals/group. B (bone), D (dentin), EMD (enamel matrix derivative), hypo (occlusal hypofunction), P (pulp), PDL (periodontal ligament).

Osteocalcin is also a protein involved in bone homeostasis. Occlusal hypofunction resulted in a reduced synthesis of osteocalcin, which could only be slightly increased by treatment with EMD so that the level of the normal occlusion group was not regained (Figure 5a–c). Overall, despite the observed negative effect of occlusal hypofunction on osteocalcin, the differences between the groups were not significant (Figure 5a). Nevertheless, moderate to very high intensity categories were only observed in the normal occlusion group, whereas low and very low intensities were only found in the two occlusal hypofunction groups (Figure 5b).

## 3. Discussion

In the present study, we investigated the effects of occlusal hypofunction on alveolar bone healing using an established fenestration defect model in rats [63,64]. Occlusal hypofunction impaired alveolar bone healing, but this could be compensated for, although not completely, by the application of a regenerative material. These results suggest that normal occlusal loading is beneficial for alveolar bone healing, and the effect of normal occlusal loading appears to be similarly critical for alveolar bone healing as the regeneration-promoting potential of EMD.

Although some studies have addressed the role of occlusal hypofunction in preclinical studies, we are not aware of any study to date that has addressed the effect of occlusal hypofunction on alveolar bone healing during regenerative therapy with EMD. Ishida et al. demonstrated that occlusal hypofunction leads to atrophic changes in the gingiva of rats [65]. At the histological level, a disorientation of collagen fibers was observed. In addition, connective tissue fibroblasts increased and epithelial intercellular gaps were enlarged. At immunohistochemical level, connective tissue growth factor and lysyl oxidase showed increased expression in the hypofunctional group [65]. Overall, therefore, occlusal function has been shown to be an important regulatory factor in the maintenance and remodeling of periodontal structures, supporting our own observations in the present study. Shimizu et al. also found that hypofunction leads to atrophic changes in the periodontal ligament (PDL) and alveolar bone in rats [66]. The researchers found that the PDL was narrowed in the hypofunction group. In particular, the volume of the PDL and the volume ratio of alveolar bone to total tissues in the region of interest (ROI) were significantly lower in the hypofunctional group. In addition, the structure of the alveolar bone was thinner and less continuous in the hypofunction group [66]. These data are consistent with our results, showing less bone healing of the fenestration defect in the hypofunction group. The effects of hypofunction on interradicular alveolar bone and PDL were studied by Kasahara et al. [67]. They found that bone volume per tissue volume in µCT decreased with hypofunction. They also observed that the hypofunction group had a smaller PDL volume [67]. Thus, these results further support our findings that hypofunction has a negative impact on alveolar bone healing. The study by Kunii et al. came to similar conclusions when they examined bone density in occlusal hypofunction and its rehabilitation in a rat model [68]. The researchers found that hypofunction led to decreased bone mineral density in both cancellous and cortical bone. This decrease was reversed after the restoration of occlusion [68]. These results are in line with our findings and show that normal occlusion is important for the healing of the alveolar bone. King and Hughes investigated the effects of occlusal loading on ankylosis, bone, and cement formation in the presence and absence of BMP-2 in an in vivo rat fenestration model [63]. Fenestration defects were treated with a collagen membrane with and without BMP-2. Interestingly, this study found that bone regeneration was faster in the hypofunctional group after 10 days, regardless of whether BMP-2 was administered. Different time periods may explain the contradictory observations in this study compared with the above studies and our own study. In addition, the collagen membranes were always placed in addition to the regenerative material, i.e., BMP-2. Nevertheless, these authors conclude that occlusal loading is an important stimulus for both remodeling and maintenance of the PDL space during early wound healing [63].

In our study, after a healing period of four weeks, we investigated whether occlusal hypofunction has an impact on bone healing and whether this effect is modulated by the application of a regeneration-promoting material, i.e., EMD. The animals suffering from occlusal hypofunction showed delayed bone regeneration compared to the group with normal occlusion. The application of EMD could partially, but not completely, compensate for the negative effects of occlusal hypofunction on bone healing. Furthermore, we found a lower synthesis of periostin in the hypofunction group. This effect was more than reversed by the application of EMD. Periostin is a molecule with multiple properties and functions. It is important for the maintenance and regeneration of periodontal tissues and is produced in larger quantities during the healing of bone fractures [57]. At the cellular level, this molecule is involved in various functions, such as cell migration, recruitment, adhesion, and proliferation [57]. By promoting the regulation and migration of fibroblasts and osteoblasts, bone remodeling and PDL are influenced [57]. Periostin thus appears to be involved in various phases of bone healing and regeneration [57]. The in vitro study by Wu et al. showed that periostin accelerates the migration, proliferation, and osteogenic differentiation of human PDL mesenchymal stem cells [69]. Kasahara et al. have shown that the expression of periostin in PDL is reduced in the presence of occlusal hypofunction [67]. In vitro data have revealed that periostin deficiency leads to the poor adhesion of osteoblasts to the bone matrix, which impaired osteoblast differentiation [57]. In an in vivo study using Wistar rats, the effects of hypofunction on PDL and changes in periostin gene and protein levels were analyzed. The results of the study demonstrated that hypofunction was associated with structural changes in the PDL, such as reduced PDL width and a decrease in the number and thickness of PDL fibers. Moreover, periostin was downregulated in the hypofunction group as compared with the control [70]. In the above studies, periostin was investigated in PDL, as this molecule is mainly expressed and produced by PDL cells. However, periostin is also known to be present in alveolar bone [57,60]. As our study aimed to examine the effects of occlusal hypofunction on alveolar bone healing, we focused on the production of proteins associated with bone regeneration. In our study, it was also observed that a lack of biomechanical loading, i.e., occlusal hypofunction, resulted in a reduced synthesis of periostin, suggesting that normal occlusion exerts a positive effect on several tissue structures of the periodontium.

Periostin deletion leads to the maladhesion of osteoblasts to the bone matrix and impairs their differentiation into mature osteoblasts, as evidenced by the downregulation of type I collagen, osteocalcin, osteopontin, and alkaline phosphatase expression in vitro [60]. Therefore, in addition to periostin, the regulation of osteopontin and osteocalcin syntheses was also evaluated in our study. Occlusal hypofunction also resulted in lower osteopontin synthesis, and this inhibitory effect of occlusal hypofunction was compensated by EMD. Additionally, occlusal hypofunction also tended to inhibit osteocalcin production. Osteopontin is a negatively charged, glycosylated phosphoprotein found in many tissues. Osteopontin is produced in bone by both osteoblasts and osteoclasts. This molecule seems to be involved in various functions within the inflammatory and tissue repair cascade [58]. Osteocalcin is a bone protein produced mainly by osteoblasts and, to a lesser extent, by odontoblasts. It is thought to inhibit bone mineralization, with recent studies suggesting an even broader role for osteocalcin [59]. Several studies have addressed the expression and protein synthesis of osteopontin and osteocalcin in periodontium. Ivanovski et al. investigated the expression of hard-tissue-associated proteins, including osteopontin and osteocalcin, in vitro and in histological sections [71]. Osteopontin and osteocalcin were detected in all components of the periodontium, i.e., gingiva, PDL, cementum, and bone [71]. An investigation of the alveolar bone healing after the extraction of a maxillary incisor in rats showed that both bone volume and trabecular thickness increased over time. Furthermore, the osteopontin and osteocalcin expressions were upregulated after tooth extraction, indicating that these molecules play an important role in alveolar bone healing [72]. In relation to bone healing, Lekic et al. immunohistochemically investigated osteopontin expression in a rat model with periodontal window wounds. They also found that osteopontin is involved in bone healing [73]. These studies highlight the important role of osteopontin and osteocalcin in periodontal healing. Our results show, for the first time, that these important bone-associated molecules are reduced in the absence of occlusal loading.

We also investigated the activity of osteoclasts in the alveolar bone using TRAP staining. Surprisingly, analysis of the stained histological sections revealed that there were no significant differences in the total number of TRAP+ cells between the studied groups. The fact that the number of osteoclasts was approximately the same in the groups may be due to the fact that, in our model, we did not analyze periodontal destruction, i.e., resorption, but the healing process.

Two main models have been described in the literature to study the effects of occlusal hypofunction. The occlusal hypofunction of a tooth can be induced by extracting the antagonist or by increasing the occlusal load on the adjacent teeth [63,65,66,67,68]. In our study, the antagonist was extracted to establish occlusal hypofunction. This model has the advantage that the possible effect of overloading the adjacent teeth on the wound healing area under investigation is minimal.

In addition to reducing pathogenic microorganisms and inflammatory and proteolytic processes, periodontal therapy involves restoring lost periodontal structures through regenerative therapy whenever possible. Preclinical studies have shown that EMD and BMP-2 promote alveolar bone healing [74,75]. For example, EMD increased osteoprotegerin (OPG) and decreased receptor activator of nuclear factor-kappa B ligand (RANKL), resulting in a better OPG/RANKL ratio in vitro [74]. In addition, BMP-2 has been shown to have a stimulatory effect on alveolar bone healing in animal studies [75]. Various animal models are available to study alveolar bone healing, with the fenestration model used in this study being well established [63,64]. The aim of our in vivo study was also to evaluate the effects of occlusal hypofunction on bone healing during regenerative therapy. Both histologically and immunohistochemically, the use of EMD generally resulted in a reduction in the negative effects of occlusal hypofunction. The application of EMD was able to partially compensate for occlusal hypofunction, although not completely in most cases. This confirms the regeneration-promoting effects of EMD reported in the literature [24,25,28,29]. EMD consists of enamel matrix proteins derived from porcine tooth buds [24,76]. EMD regulates numerous cell functions associated with periodontal regeneration. For example, EMD increases cell proliferation and migration, matrix molecule synthesis, angiogenesis, and mineralization [24,77]. EMD has been shown to stimulate the synthesis of periostin, osteopontin, and osteocalcin during regenerative therapy [30,78,79]. In addition, the regeneration-promoting effects of EMD could also be mediated by its anti-inflammatory properties [31,35]. Furthermore, the antibacterial properties of the EMD vehicle used for the clinical application could contribute to the regenerative effects [32,48,80]. The regenerative potential of EMD has been clinically proven by a lot of studies [81,82]. Histological human sections clearly prove that periodontal regeneration can take place after the application of EMD [28,29]. Future studies could also focus on the interactions between occlusal hypofunction and other regenerative materials. It would also be interesting to analyze whether, and to what extent, occlusal hyperfunction modulates bone healing in the presence or absence of EMD or other regenerative materials.

Our study has some limitations. One limitation is that the effect of occlusal hypofunction on alveolar bone healing was investigated only 4 weeks after the induction of the fenestration defect. The healing process after regenerative surgical periodontal treatment is largely completed in the first 2–3 postoperative weeks, followed by further tissue maturation to adapt to the functional needs of the masticatory environment [83]. We chose to examine bone regeneration after 4 weeks, so that tissue healing is complete but not yet fully adapted to the functional conditions of the oral and maxillofacial systems. This decision is consistent with many other published studies using the same surgical model. In most of these studies, animals were sacrificed between 21 and 30 days after surgery [33,63,64,84,85,86,87]. Nevertheless, analysis at different time points during healing would provide more detailed information on the effects of occlusal hypofunction on alveolar bone healing. By including additional evaluation time points, e.g., at 10 and 21 days postoperatively, serial analysis of periostin, osteopontin, and osteocalcin would have been possible and may have revealed different kinetics of these molecules in relation to remodeling. However, a higher number of animals would have to be sacrificed for these additional time points. The present study also has technical limitations. Preparation of the histologic sections was associated with technical difficulties related to the transverse plane. In some cases, the blocks had to be melted and reembedded until the correct plane was achieved. Histological slides are always limited, so only a restricted number of stainings could be performed. The application of calcein would provide additional information on the mineralization rate, and therefore on the bone regenerative processes. In the present study, only bone formation was evaluated. Future studies or evaluations should also investigate the influence of hypofunction under normal or regenerative conditions on other components of the periodontium, e.g., PDL and cementum.

In a fenestration defect model in rats, we demonstrated that occlusal hypofunction compromised alveolar bone healing and that the application of a regenerative material partially compensated for this inhibition. These results suggest that normal occlusal loading, but not occlusal hypofunction, is beneficial for alveolar bone healing. Adequate occlusal loading appears to be as beneficial for alveolar bone healing as the regenerative effects of EMD.

## 4. Materials and Methods

### 4.1. Animal Model

After receiving approval for this animal study from the University of Bonn and the local authorities (Landesamt für Natur, Umwelt und Verbraucherschutz Nordrhein-Westfalen; AZ 85-51.04.2010.A394), four-week-old Wistar rats were obtained from the Charles River Laboratories (Sulzfeld, Germany). All animals were kept in the animal facility of the University of Bonn according to institutionally approved protocols. All applicable international, national, and/or institutional guidelines for the care and use of animals have been followed. Per group, five animals (15 animals in total) were maintained under stable environmental conditions at a room temperature of 21 °C and humidity of 35% under a 12 h day–night cycle. All groups of animals were provided with water and food (sniff, Soest, Germany) ad libitum. Each animal group underwent a different experimental treatment: (1) normal occlusion with an untreated fenestration defect, (2) occlusal hypofunction with an untreated fenestration defect, and (3) occlusal hypofunction with a fenestration defect treated with EMD (Emdogain^®^, Straumann, Basel, Switzerland).

### 4.2. Surgical Procedures

Animals were treated under general anesthesia by using ketamine 10% (75 mg/kg) and medetomidine (0.5 mg/kg) intraperitoneally. During surgery, animals were placed on a warm surface to prevent hypothermia and the airways were kept clear.

To induce occlusal hypofunction, the first molar of the upper right jaw was extracted. In brief, local anesthesia was performed in the vestibule above the first molar with articaine 1:200,000 dissolved in saline according to the body weight of the animals. The PDL was loosened by a fine dental probe. The tooth was mobilized by using a small Heidemann spatula. The tooth was extracted with the help of a small artery clamp. The wound was smoothly compressed and, if needed, treated with a suture (4.0 Vicryl, Ethicon, Johnson & Johnson Medical, Norderstedt, Germany).

In order to investigate the influence of occlusal hypofunction on alveolar bone regeneration, a fenestration-type defect was created in the first molar of the lower right jaw corresponding to the extracted tooth of the upper jaw. The fenestration-type defect was created as described before [58]. Briefly, after shaving and disinfecting the skin on the right side of the lower jaw with liquid povidone iodine, a 2 cm incision was made at the inferior border of the mandible using a 15c scalpel (Hu-Friedy, Frankfurt am Main, Germany). The superficial fascia, the underlying masseter muscle and the periosteum were severed from the bone and the mandible was exposed. The tissue was carefully prepared with a rasp until the oral mucosa was identified. The preparation was terminated to avoid injury to the adherent keratinized gingival margin at the upper edge of the intraoral surgically created access chamber. With a slow-speed rose bur and by using saline irrigation, the buccal bone of the first molar of the lower right jaw was removed until the root surface was visible. The fenestration defect was standardized with an approximate height and depth of 2 mm and a horizontal dimension of 4 mm. The dimensions of the fenestration defect were assessed using a standardized periodontal probe (UNC 15, Hu-Friedy) during surgery. The upper margin of the bony defect was 1 mm from the crestal bone of the first molar. The exposed first molar root was freed from PDL, cementum, and dentin. Root surface was rinsed with saline and subsequently conditioned with ethylenediaminetetraacetic acid (EDTA, PrefGel^®^, Straumann) for 2 min according to the manufacturer’s instructions. After 2 min of conditioning, the surface was thoroughly rinsed with saline. Animals of the group (3) were treated with EMD (Emdogain^®^, Straumann). EMD was applied using a sterile syringe so that the complete defect was covered. A double layer suture (muscle and skin) of absorbable suture material (4.0 Vicryl, Ethicon, Johnson & Johnson Medical) was placed for wound closure. After surgery, all animals received carprofen (5 mg/kg) as analgesia. Every other day after surgery, and for a whole week, wound healing and the general condition of the animals were monitored. Weight, swelling, and wound healing were controlled and documented. None of the animals showed local or general complications, such as unnatural weight loss or wound infection, after surgery. Four weeks after surgery, all animals were sacrificed, and skulls were removed and stored in 4% phosphate-buffered formaldehyde (Merck, Darmstadt, Germany) for one week for fixation.

### 4.3. Histological Preparation and Staining

The histological preparation and staining of the skulls were carried out as previously described. In short, the skulls were decalcified for 12 weeks in a 10% EDTA solution (EMD Millipore, Billerai, MA, USA). Skulls were further prepared so that the right mandible was separated and embedded in paraffin after dehydration in an ascending ethanol series. The jaws were oriented in the paraffin blocks in such a manner that transversal sections with a 2.5 µm thickness were cut. Sections were mounted on glass slides (Engelbrecht, Edermünde, Germany) and dried overnight at 37 °C. Whether the transverse plane was achieved was checked by staining the slides according to hematoxylin and eosin (H&E; Merck) using a standardized protocol [88] and by light microscopic examination. In case of incorrect plane, blocks were melted and newly embedded until the correct plane was reached and all slides including the region of interest could be cut. All sections from the region of interest were collected and used for further staining. In order to recognize the coronal, middle, and apical level of the defect, every tenth histological section was stained according to H&E and controlled under a light microscope. One section of each level (coronal, middle, apical) from all 15 animals was included in the analyses. Identification of osteoclasts was achieved by staining the three serial sections, corresponding to the ones used for H&E staining, using TRAP staining (Sigma-Aldrich, Taufkirchen, Germany) following the manufacturer’s instructions.

### 4.4. Immunohistochemical Staining

After deparaffinization and dehydration, slides were treated by blocking endogenous peroxidase with 0.3% methanol (Merck)/30% H_2_O_2_ (Merck) for 10 min in the dark. For periostin staining, sections were pretreated with pepsin for 20 min at 37 °C. Subsequently, sections were pre-blocked with 1 × tris-buffered saline (Merck)/4% bovine serum albumin (Merck) for 1 h at room temperature and incubated in a humid chamber overnight at 4 °C with a rabbit polyclonal antibody against periostin with a concentration of 1:300 (ab14041, Abcam, Cambridge, UK). For the detection of osteopontin and osteocalcin, no pre-treatment of sections was needed. Sections were immediately incubated with a rabbit polyclonal antibody against osteopontin with a concentration of 1:300 for 1 h at room temperature (ab8448, Abcam) and with a mouse monoclonal antibody against osteocalcin with a concentration of 1:1,200 overnight in a humid chamber at 4 °C (ab13418, Abcam). Afterwards, sections were rinsed and incubated at room temperature with a goat anti-rabbit IgG-HRP secondary antibody or a goat anti-mouse IgG-HRP secondary antibody (Dako, Hamburg, Germany) for 30 min. Peroxidase activity was visualized by incubation with 3,3-diaminobenzidine chromogen (Thermo Fisher Scientific, Dreieich, Germany). Finally, sections were rinsed and counterstained for 30 s with Mayer’s hematoxylin (Merck).

### 4.5. Histomorphometric Evaluation

Images of all sections were captured with the Axioskop 2 microscope and AxioVision 4.7 software (Carl Zeiss, Jena, Germany) at 10× magnification. The region of interest (ROI) was selected and defined as described before (Figure S2) [64]. In brief, the root of the first molar and the fenestration defect formed the ROI. The ROI was defined within a standardized quadrant, which included root, PDL and bone of the buccal site of the first molar. The standardized quadrant was placed on each image and the area was cut off within the limits of the quadrant. The new images were saved and used for further evaluation. Analyses were conducted with the open-source programs ImageJ and Fiji plug-in [89,90]. Within the ROI of H&E-stained slides, bone was manually selected and quantified. The percentage ratio of bone area to total area of the ROI was calculated and used for statistical analysis. TRAP+ cells were manually counted within the ROI. For immunohistochemistry, the intensity of staining within the ROI was quantified by the Fiji plug-in. Values were divided into five categories, defined by the respective quintiles. Thus, values of intensity were categorized as follows: 1: very low, 2: low, 3: moderate, 4: high, and 5: very high. They were subsequently analyzed, as described previously [64].

### 4.6. Statistical Analysis

For statistical analysis, the IBM SPSS Statistics Software (Version 27, IBM SPSS, Chicago, IL, USA) was applied. Normal distribution was examined using the Kolmogorov–Smirnov test and the homogeneity of variances was examined using Levene’s test. Mean values and standard errors of the means were calculated. One-sided Mann–Whitney U test and ANOVA with post-hoc LSD were applied to detect statistically significant differences between the groups (*p* < 0.05).

## Data Availability

Data sharing is not applicable to this article.

## Supplementary Materials

The following supporting information can be downloaded at:https://www.mdpi.com/article/10.3390/ijms24119744/s1.

## Abbreviations

B	bone
BMP-2	bone morphogenetic protein 2
D	dentin
EDTA	ethylenediaminetetraacetic acid
EMD	enamel matrix derivative
H&E	hematoxylin and eosin
OPG	osteoprotegerin
P	pulp
PDL	periodontal ligament
RANKL	receptor activator of nuclear factor-kappa B ligand
ROI	region of interest
TRAP	tartrate-resistant acid phosphate
