# Supplementary material for: Influence of Occlusal Hypofunction on Alveolar Bone Healing in Rats
Source: Int J Mol Sci. 2023 Jun 5;24(11):9744. doi: 10.3390/ijms24119744 (PMC10253992; doi:10.3390/ijms24119744)
Supplement: Supplementary file 1 [file ijms-24-09744-s001.zip › ijms-2221411-supplementary.pdf]

International Journal of
Molecular Sciences

MDPI

Article

Influence of occlusal hypofunction on alveolar bone healing in rats

Anna Damanaki [1,*], Svenja Beisel-Memmert [2], Marjan Nokhbehsaim [3], Ali Abedi [1], Birgit Rath-Deschner [2], Andressa V.B. Nogueira [1] and James Deschner [1]

[1] Department of Periodontology and Operative Dentistry, University Medical Center, University of Mainz, 55131 Mainz, Germany

[2] Department of Orthodontics, Center of Dento-Maxillo-Facial Medicine, University of Bonn, 53111 Bonn, Germany

[3] Section of Experimental Dento-Maxillo-Facial Medicine, Center of Dento-Maxillo-Facial Medicine, University of Bonn, 53111 Bonn, Germany

* Correspondence: adamanak@uni-mainz.de; Tel.: +49-(0)-61-3117-5419

Abstract: The aim of this in vivo study was to investigate the effect of occlusal hypofunction on alveolar bone healing in the absence or presence of an enamel matrix derivative (EMD). A standardized fenestration defect over the root of the mandibular first molar in 15 Wistar rats was created. Occlusal hypofunction was induced by extraction of the antagonist. Regenerative therapy was performed by applying EMD to the fenestration defect. The following three groups were established: (a) normal occlusion without EMD treatment, (b) occlusal hypofunction without EMD treatment, and (c) occlusal hypofunction with EMD treatment. After four weeks, all animals were sacrificed, and histological (hematoxylin and eosin, tartrate-resistant acid phosphatase) as well as immunohistochemical analyses (periostin, osteopontin, osteocalcin) were performed. The occlusal hypofunction group showed delayed bone regeneration compared to the group with normal occlusion. The application of EMD could partially, but not completely, compensate for the inhibitory effects of occlusal hypofunction on bone healing, as evidenced by hematoxylin and eosin and immunohistochemistry for the aforementioned molecules. Our results suggest that normal occlusal loading, but not occlusal hypofunction, is beneficial to alveolar bone healing. Adequate occlusal loading appears to be as advantageous for alveolar bone healing as the regenerative potential of EMD.

Keywords: bone healing; occlusal hypofunction; fenestration; animals, periodontitis

Supplementary material

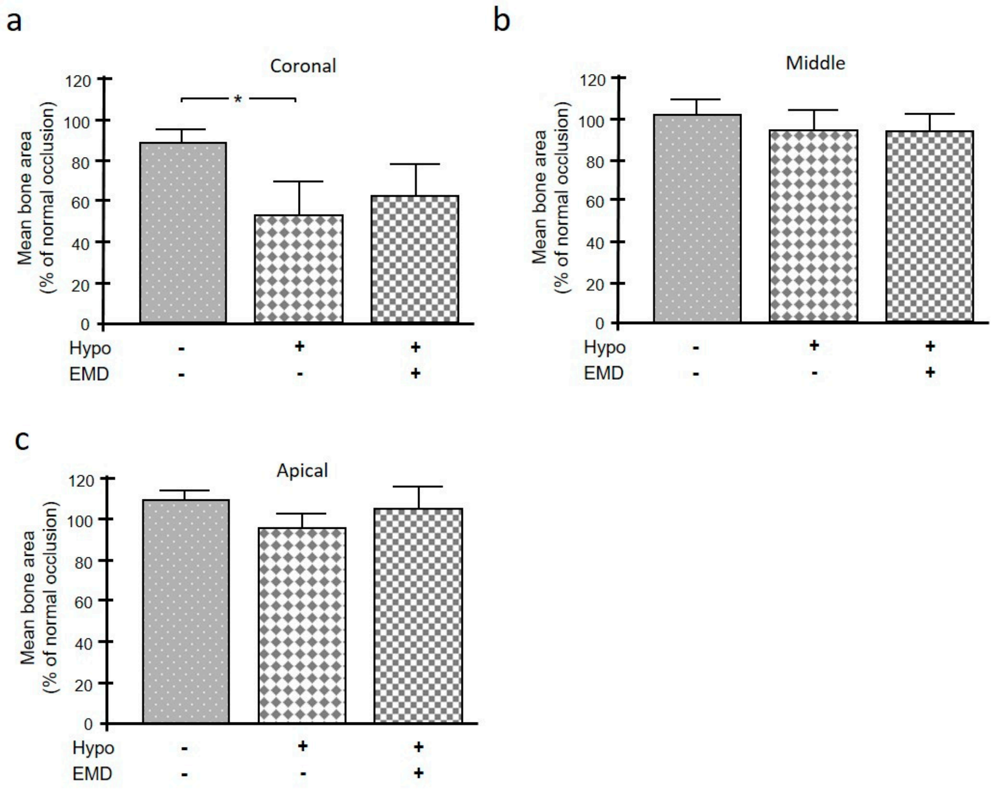

Figure S1. Effect of occlusal hypofunction on alveolar bone healing in the presence and absence of EMD at the coronal (**a**), middle (**b**) and apical (**c**) level. Bars show mean ± SEM; n=5 animals/group; * significant ($p<0.05$) difference between groups. Coronal (coronal level), Middle (middle level), Apical (apical level).

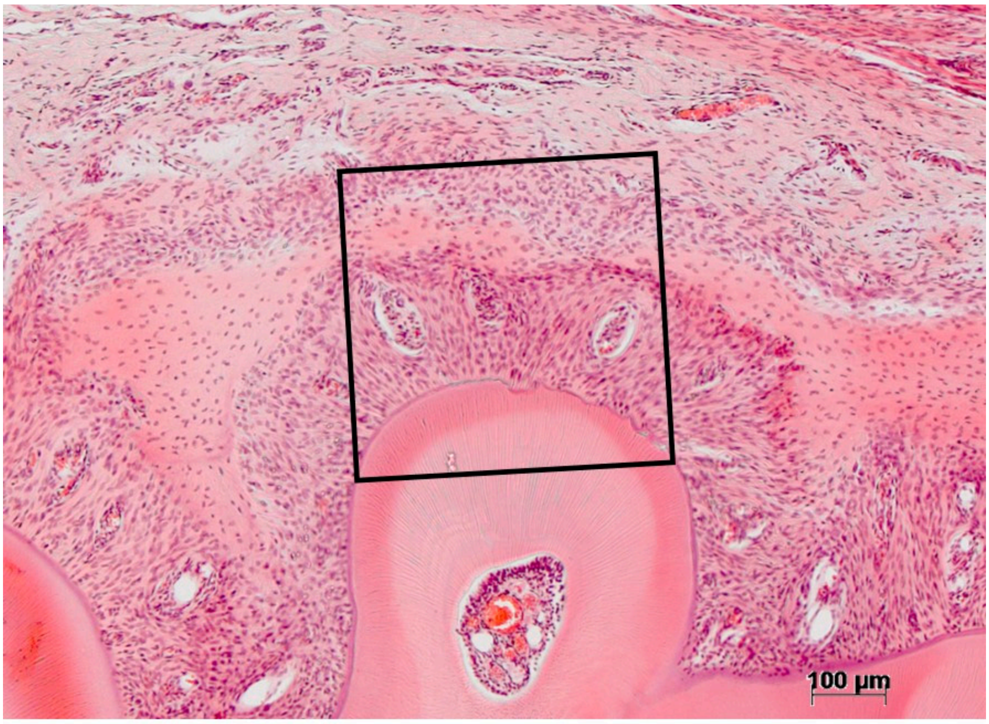

Figure S2. Representative image of H&E stained tissue sections with the standardized quadrant, which defines the ROI.
